# 560 Is the Need for Follow up Lost in Translation?

**DOI:** 10.1093/jbcr/irae036.194

**Published:** 2024-04-17

**Authors:** Larissa Epstein, Carina Franco, Alexandra Coward, Jason Heard, Soman Sen, Tina L Palmieri, Kathleen S Romanowski

**Affiliations:** UC Davis, Menlo Park, CA; University of California, Davis School of Medicine, Los Angeles, CA; UC Davis, Sacramento, CA; University of California - Davis Hosp, Sacramento, CA; UC Davis, Shriners Children's Northern California, Sacramento, CA; UC Davis, Menlo Park, CA; University of California, Davis School of Medicine, Los Angeles, CA; UC Davis, Sacramento, CA; University of California - Davis Hosp, Sacramento, CA; UC Davis, Shriners Children's Northern California, Sacramento, CA; UC Davis, Menlo Park, CA; University of California, Davis School of Medicine, Los Angeles, CA; UC Davis, Sacramento, CA; University of California - Davis Hosp, Sacramento, CA; UC Davis, Shriners Children's Northern California, Sacramento, CA; UC Davis, Menlo Park, CA; University of California, Davis School of Medicine, Los Angeles, CA; UC Davis, Sacramento, CA; University of California - Davis Hosp, Sacramento, CA; UC Davis, Shriners Children's Northern California, Sacramento, CA; UC Davis, Menlo Park, CA; University of California, Davis School of Medicine, Los Angeles, CA; UC Davis, Sacramento, CA; University of California - Davis Hosp, Sacramento, CA; UC Davis, Shriners Children's Northern California, Sacramento, CA; UC Davis, Menlo Park, CA; University of California, Davis School of Medicine, Los Angeles, CA; UC Davis, Sacramento, CA; University of California - Davis Hosp, Sacramento, CA; UC Davis, Shriners Children's Northern California, Sacramento, CA; UC Davis, Menlo Park, CA; University of California, Davis School of Medicine, Los Angeles, CA; UC Davis, Sacramento, CA; University of California - Davis Hosp, Sacramento, CA; UC Davis, Shriners Children's Northern California, Sacramento, CA

## Abstract

**Introduction:**

There is limited research evaluating patients of limited English proficiency (LEP) on outcomes after burn injury. Studies have shown that patients with LEP have reduced access to care, longer admissions, are more likely to be discharged to a nursing facility and are more likely to return to the emergency department (ED). Burns require significant inpatient and outpatient coordinated care for successful discharge planning and patient education regardless of language preference. This study examines the effect of LEP on outcomes following burn injury. We hypothesize that burn patients with LEP will have longer hospital stays, fewer follow up (F/U) visits, and more ED visits after discharge.

**Methods:**

Following IRB approval, a retrospective chart review was conducted using electronic medical records for burn patients admitted from January 2018 to December 2019. Data collected includes patient demographics, burn injury, burn outcomes, preferred language, and F/U care. Analysis was conducted with SAS statistical software, version 9.4 (SAS Institute, Cary, NC, USA) using Chi-square, Fisher Exact, Spearman Correlation, Wilcoxon 2-sample and Kruskal-Wallis tests.

**Results:**

A total of 760 patients with a median age of 46 years (Interquartile range (IQR) = 26) were analyzed. Median total body surface area (TBSA) burn was 6.5 (IQR = 12) and 15% had inhalation injury. The median length of stay (LOS) was 9 days (IQR = 18) and 38 patients (5%) died. Reviewing post-burn care, 613 patients (81%) scheduled F/U, 397 patients (52.7%) attended a F/U appointment within 30 days of discharge, 85 patients (11.2%) presented to the ED, and 46 patients (6.1%) were readmitted. Only 61 patients (8%) preferred a language other than English. Among patients with LEP, Spanish was the most spoken language (5.6%), followed by Hmong (0.92%). When patients with LEP were compared with English speakers there was no difference in age (42 (22) vs. 46 (26), p=0.67), TBSA (5.5 (10.5) vs. 6.6 (11.8), p=0.32), mortality (3.3% vs. 4.7%, p=1), LOS (8 (16) vs. 9 (18), p=0.43), ICU days (2 (16) vs. 3 (15), p=0.43), inhalation injury (13.1% vs. 14.9%, p=0.87), and discharge to home (86.9% vs. 75.9%, p=0.44). Patients with LEP were more likely to have follow up scheduled at discharge (93.4% vs. 80.2%, p=0.04), attend F/U (78.7% vs. 50.7%, p=0.0005), and present to the ED after discharge (19.7% vs. 10.5%, p=0.03). There was a trend towards them being readmitted more than native English speakers (11.5% vs. 5.6%, p=0.08).

**Conclusions:**

Patients with LEP were no different from their English-speaking counterpart with respect to traditional outcome measures (LOS or mortality) but had increased healthcare utilization (F/U or return to the ED).

**Applicability of Research to Practice:**

As the number of patients with LEP increases, health care providers must be aware of the influence this has on outcomes following burn.

